# A 16-week study to compare the effect of vildagliptin versus gliclazide on postprandial lipoprotein concentrations and oxidative stress in patients with type 2 diabetes inadequately controlled with metformin monotherapy

**DOI:** 10.1186/s13098-015-0058-8

**Published:** 2015-07-11

**Authors:** Marcelo R. Nasser Hissa, Lilian Loureiro Albuquerque Cavalcante, Sergio Botelho Guimarães, Miguel Nasser Hissa

**Affiliations:** Department of Surgery, Postgraduate Program, UFC, Fortaleza, Ceara Brazil; Department of Surgery, Head, LABCEX, UFC, Fortaleza, Ceara Brazil; Department of Medicine, Head of Diabetes and Endocrine-metabolic Unit Research, UFC, Fortaleza, Ceara Brazil

## Abstract

**Background:**

Diabetes is closely linked with coronary artery disease, either by means of direct effects of hyperglycemia, or indirectly by its frequent association with dyslipidemia. Any treatment for diabetes that has beyond the capacity of reduce glycated hemoglobin, the propensity to improve lipid profile and reduce weight will bring many benefits to patients.

**Method:**

We compare the effects of vildagliptin with the gliclazide on lipid profile before and after a standardized meal test, on glycemic control and oxidative stress in diabetic patients using metformin without adequate glycemic control. This is a prospective study of 16 weeks with diabetic patients using metformin without adequate glycemic control. Patients were randomized to receive gliclazide 30–120 mg/day or vildagliptin 100 mg/day.

**Results:**

36 patients were randomized, with no loss of follow up. Regarding the lipid profile the difference observed at the end of the study was a higher HDL level in the vildagliptin group compared with gliclazide fasting (62.3 vs. 51.3 mg/dL, *p* = 0.021) and postprandial (62.9 vs. 51.1 mg/dL, *p* = 0.015). We also observed a variation of negative weight (decrease the end compared to the beginning) of the vildagliptin and a positive (increase) in the gliclazide (−0.3 vs. +1.4 Kg, *p* = 0.048). The decrease in A1c was lower in the vildagliptin group compared to gliclazide (−1.7 vs.−2.3 %, *P* = 0.031), however there was no difference in the number of patients reaching target glycated hemoglobin <7 % (50 vs. 61.1 %, *p* = 0.738). Only the group of vildagliptin presented at the end of the study compared to the beginning, decreased insulin values (599.6 vs.705, 59 pg/ml, *p* = 0.021), glucagon (46.6 vs.65, 2 pg/ml, *p* = 0.004) and the marker of oxidative stress TBARS (8.0 vs. 9.0 nmol MDA/ml, *p* = 0.035).

**Conclusion:**

Vildagliptin showed some advantages in addition to metformin in relation to addition of gliclazide. Patients treated with vildagliptin had a higher HDL at the end of the study, less variance in weight, reduced insulin and glucagon as well as reduction of oxidative stress.

## Introduction

Diabetes is a devastating disease that currently affects more than a billion people worldwide [1, 2]. Patients with diabetes have a significantly increased risk of morbidity and mortality associated with cardiovascular disease and stroke, causing 75 % of all deaths [3–5]. Furthermore, type 2 diabetes mellitus (T2DM) is usually associated with other factors like hypertension, dyslipidemia, obesity and accelerated atherosclerosis risk that contributes to an even higher morbidity and cardiovascular mortality [6].

Free radicals are formed disproportionately in diabetes by glucose oxidation, nonenzymatic glycation of proteins, and the subsequent oxidative degradation of glycated proteins. Abnormally high levels of free radicals and simultaneous decline of antioxidant defense mechanisms can lead to damage of cellular organelles and enzymes, increased lipid peroxidation, and development of insulin resistance. Conversely insulin resistance is associated with atherogenic dyslipidemia, with the postprandial hyperlipidemia playing an important role in this state [7, 8]. It is well known that impaired clearance from the circulation of intestinally-derived lipoprotein particles and their lipid content contributes to postprandial hyperlipidemia in those with insulin resistance and T2DM [9].

Gliclazide is a second generation sulphonylurea (SU) most prescribed in many countries. It has advantage over others SUs because of its selectivity. Individual SUs express a different selectivity for pancreatic and myocardial SU receptors; gliclazide seems the most selective with respect to pancreatic receptor stimulation, and thus showing a cardiovascular benefits over others SUs [10].

Vildagliptin is a compost of the DPP-4 inhibitor drug class. It is a highly-selective substrate for the DPP-4 catalytic site with a slow reaction rate, which blocks the usually rapid degradation of GLP-1 and glucose-dependent insulinotropic peptide (GIP) [11, 12]. Vildagliptin increases plasma level of GLP-1 and GIP, which improves the sensitivity of the β- and α-cell to glucose; there are also extra-pancreatic effects which contribute to improved insulin sensitivity and attenuate weight gain. In clinical studies, vildagliptin has been shown to reduce HbA1c, fasting plasma glucose (FPG) & postprandial plasma glucose levels. Furthermore decreased postprandial glycemic excursion might reduce the oxidative stress markers and improve postprandial hyperlipidemia [13].

Results from clinical studies to date indicating that dipeptydyl peptidase-4 (DPP-4) inhibitors reduce total cholesterol are inconsistent. Based on these evidence, this research was designed to evaluate the effect of vildagliptin on the impact of DDP-4 inhibition on lipoprotein metabolism by examining the effects of vildagliptin on postprandial lipid levels and oxidative stress. We hypothesized that in T2DM, treatment with vildagliptin would improve lipoprotein levels and oxidative stress more pronounced than treatment with gliclazide.

## Subjects and methods

### Study design

This is a 16-week open label randomized prospective study conducted at the Diabetes Research Center/Department of Internal Medicine, Federal University of Ceara, Brazil.

The study protocol was approved by the local Ethical Committee (Comite de etica em pesquisa/ Hospital universitario Walter Cantidio) and all eligible candidates had to provide signed informed consent before enrolling in the study.

### Participants

We enrolled 36 patients with type 2 diabetes who had been inadequately treated with metformin for at least 3 months (≥1,000 mg/day) expressed as HbA_1c_ > 7,5 %. Eligibility criteria included men or women 18–70 years of age who were on metformin treatment for ≥3 months on stable dose (≥1000 mg/daily) and body mass index between ≥ 22 and ≤ 40 kg/m2. The exclusion criterias were following: (1) pregnant or nursing women; (2) use of any anti-diabetic treatment within 3 months prior to visit 1 other than metformin; (3) chronic (>7 consecutive days) oral, parenteral or intra-articular corticosteroid treatment within 8 weeks prior to Visit 1; (4) history or evidence of major hepatopathy (aspartate aminotransferase or alanine aminotransferase activities > 2.5 times the upper limit of normal); (5) ischemic heart disease or cerebrovascular disease; (6) creatinine level > 0.133 mmol/L; (7) major diabetes complications (chronic renal insufficiency, proliferative retinopathy and stroke); (7) extreme dyslipidemia, such as familial hypercholesterolaemia; (8) use of antilipemic drug; (9) and a recent history of alcohol or drug abuse.

### Treatment protocol

**Fig. 1:**
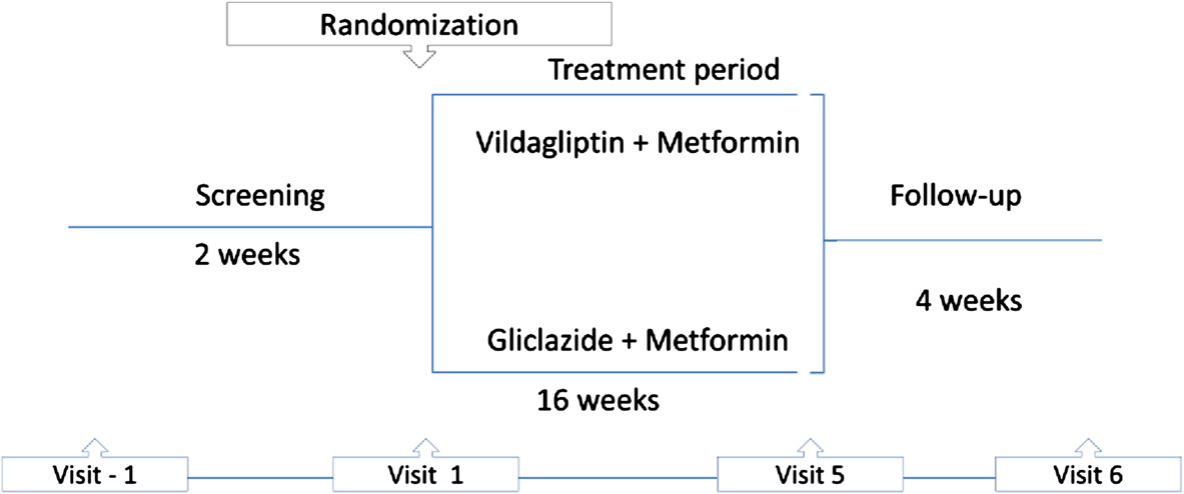
Study design

The study consisted of a 2-week screening period and a 16-week treatment period with either vildagliptin or gliclazide plus metformin, followed by 4 week on follow up. At the initial interview, height, body weight, vital signs and physical examination were performed. Fasting blood samples were performed to assure the inclusion and exclusion criterias (A1c, fast glucose, hepatic function test, serum blood urea nitrogen and creatinine and hematology). At visit 1 (2 weeks later) selected patients were randomly assigned to either vildagliptin or gliclazide group. Participants assigned to vildagliptin group were instructed to take one capsule (50 mg) before their morning meal and before dinner. Participants assigned to gliclazide group were instructed to take the dose of gliclazide before the morning meal. It was allowed to titrate gliclazide to a maximum of 120 mg/daily. Compliance was assessed by pill counting. Blood was also collected at visit 1 (week zero) for the assessment of, A1c, fast glucose, lipid profile (total cholesterol, triglyceride, HDL cholesterol, oxidative stress markers [reactive substances to thiobarbituric acid (TBARS), total antioxidant status (TAOS)]; Insulin, C- peptide, glucagon, GLP-1 and GIP. All laboratory tests were repeated in visit 5 (week 16) (Fig. 1).

#### Meal test

Before randomization (week 0) and at week 16 after randomization, a standardized meal test (*ensure plus®*) was given. It consisted of a milkshake containing 13 g protein (16.7 %), 40 g carbohydrate (53.8 %) and 13 g lipid (29.5 %) per 300 Kcal. The patients thereby fasted overnight and an intravenous catheter was inserted into a forearm vein for blood sampling. Study drug was administered 30 min before consumption of a standardized breakfast meal. The meal was consumed within 5 min, and blood samples were drawn before the meal (fasting state) and 3 h after the meal (postprandial state) for the assessment of lipid profile.

### Assays and methods of dosage

Plasma glucose was assayed by glucose-oxidase method (Dimension RxL Max Integrated Chemistry System – Siemens AG, Erlangen, Germany); Patients were also instructed to monitor their capillary blood glucose by using a blood glucose monitor (Accu-Chek Performa – Roche Diagnostica Brasil LTDA, São Paulo, SP, Brazil). This was done every 3 to 5 days before each visit (visit 2, 3 and 4), seven times a day (before and 2 h after breakfast, lunch, dinner, and before supper).

Glycated hemoglobin (A1c) was determined by High-performance liquid chromatography. Total cholesterol (TC), triglycerides (TG), HDL-cholesterol (HDL), blood urea nitrogen (BUN), creatinine concentrations and hepatic function tests were determined using enzymatic analysis (Hitachi Modular P800 Chemistry Analyzer – Roche Diagnostics, Indianapolis, IN, USA). Thiobarbituric Acid Reactive Substances (TBARS) and antioxidant state (TAOS) were measured with the QuantiChromTM Antioxidant Assay Kit (DTAC-100) (BioAssay Systems, Hayward, CA, USA). Insulin, Glucagon, C-Peptide, glucagon like peptide-1 (GLP-1) and gastric inhibitory polypeptide (GIP) were measured with the Human Metabolic Hormone Magnetic Bead Panel 96 Well Plate Assay (EMD Millipore Corp., St. Charles, MO, USA).

### Statistical analysis

In order to characterize the population of each group and compare some characteristics, descriptive analysis of frequency measurements was performed when the variable was qualitative; mean and standard variations when the variable was quantitative. In the comparison of qualitative independent variables the chi-square Pearson test was used. The quantitative variables were tested for normality by the Shapiro-Wilk test. For independent variables, in the presence of normality, the homogeneity of variance of the groups was evaluated using the Levene’s test. Given the homogeneity, the difference between groups was tested by applying ANOVA. In case of failure to evidence the homogeneity by Levene’s test, the differences between the independent variables were calculated using the Welch test. The distinction between quantitative variables without normality was verified using the Mann–Whitney test. In the case of paired variables that were proved normality, inequalities between groups were analyzed by paired t test. If there was normality, the test used to compare this type of variable was the Wilcoxon. The exact significance was evaluated by two-tailed test.

## Results

The study enrolled 36 patients, 18 randomized to Gliclazide group (mean daily dose 86.8 ± 28.1 mg) and 18 for the Vildagliptin. There was no loss of follow up of any of the patients. Except for weight, no differences in demographic were observed between the two groups at baseline (Table 1).

**Table 1:** Patient demographics and baseline characteristics (randomized population)

Demographic	Gliclazide (*n* = 18)	Vildagliptin (*n* = 18)
Age (years)	55.4 ± 11.3	59.4 ± 8.9
Sex (number of men)	6	12
BMI (kg/m2)	30.68 ± 4.3	29.35 ± 4.3
Duration of diabetes (years)	4.4 ± 2.6	4.3 ± 2.7
Duration of previous metformin treatment (months)	All patients were on metformin treatment for ≥3 months on stable dose (≥1000 mg/daily)	
Metformin daily dose (mg)	1457 ± 373	1584 ± 528
Gliclazide daily dose (mg)	86,80 ± 28,10	-

Data are means ± SD unless otherwise indicated

There were no differences between the two groups at baseline for biochemical tests. This is also true for lipid profile before and after a standardized meal test concerning total cholesterol, HDL-cholesterol and triglycerides (Table 2).

**Table 2:** Comparision of laboratory tests and weight between the two groups before and after treatment

	Pretreatment			Post treatment		
	Week 0			Week 16		
Parametes	Gliclazide	Vildagliptin	*P*	Gliclazide	Vildagliptin	*P*
Fasting TC (mg/dL)	195.2 ± 26.9	190.1 ± 35.8	0.636	194.0 ± 32.2	192.6 ± 36.4	0.904
Postprandial TC (mg/dL)	193.1 ± 25.9	186.8 ± 34.8	0.544	191.3 ± 32.9	192.8 ± 37.2	0.899
Fasting HDL (mg/dL)	51.1 ± 10.0	60.0 ± 18.9	0.086	51.3 ± 10.4	62.3 ± 15.6	0.021
Postprandial HDL (mg/dL)	50.2 ± 9.3	59.3 ± 17.9	0.150	51.1 ± 10.8	62.9 ± 16.0	0.015
Fasting Triglycerides (mg/dL)	177.3 ± 87.3	197.1 ± 116.0	0.567	205.1 ± 100.3	190.7 ± 106.2	0.635
Postprandial Triglycerides (mg/dL)	207.9 ± 89.3	218,4 ± 110.0	0.849	221.8 ± 90.1	197.4 ± 86.1	0.342
Fasting glucose (mg/dL)	179 ± 57.9	168.7 ± 31.3	0.475	131.7 ± 30.5	132.9 ± 24.8	0.896
Post prandial glucose (mg/dL)	242.83 ± 71.7	241.4 ± 69.5	0.825	191.2 ± 54.8	194.5 ± 47.4	0.847
A1c (%)	9.2 ± 1.2	8.6 ± 0.9	0.109	6.9 ± 1.1	6.9 ± 0.8	0.800
Weight	82.0 ± 14.6	71.4 ± 8.0	0.012	83.4 ± 14.8	71.0 ± 8.1	0.005
Weight Change (kg)	-	-	-	+1.4 ± 3.0	−0.3 ± 2.0	0.048

Data are means ± SD. *TC* total cholesterol, *HDL* high dense lipoprotein

After the 16 week study, in the stage of post-treatment, total cholesterol and triglyceride levels in fasting and postprandial remained without differences [(*p* = 0.904 and *p* = 0.899) and (*p* = 0.635 and *p* = 0.342), respectively]. However we observed an increase statistically significant in HDL cholesterol levels in fasting and postprandial (*p* = 0.021 and *p* = 0.015, respectively) in the Vildagliptin group (Table 2).

Regarding the weight, there was a difference between the groups before pretreatment. This was higher in the gliclazide compared to vildagliptin group (82.0 ± 14.6 kg and 71.4 ± 8.0 kg, respectively, *p* = 0.012) and this difference persisted after treatment (83.4 ± 14.8 and 71.0 ± 8.1 kg, respectively, *p* = 0.012). It is important to note that while in the gliclazide group, the variation of weight (post-treatment weight less pre-treatment weight) was positive (1.4 ± 3.0 kg) in the vildagliptin group it was negative (−0.3 ± 2.0 kg), with statistical significance (*p* = 0.048).

No differences were observed between the two groups in fasting and postprandial glucose levels in pretreatment (*p* = 0.475 and *p* = 0.896, respectively) as well as post-treatment (*p* = 0.825 and *p* = 0.847). A1c also showed no differences between groups in pre and post-treatment (*p* = 0.109 and *p* = 0.800 respectively) and in the number of patients reaching target glycated hemoglobin <7 % (50 vs. 61.1 % [*p* = 0.738], vildagliptin vs. gliclazide respectively).

Patients who used gliclazide showed no significant changes from pre to post-treatment regarding to insulin (*p* = 0.160), glucagon (*p* = 0.921), GLP-1 (*p* = 0.218), GIP (*p* = 0.201) and C-peptide levels (*p* = 0.193). TBAR and TAOS also showed no significant changes (*p* = 0.262 and *p* = 0.179, respectively) (Table 3).

**Table 3:** Intragroups comparation of laboratory tests before (week 0) and after treatment (week 16)

Parameters	Gliclazide			Vildagliptin		
	Week 0	Week 16	*P*	Week 0	Week 16	*P*
Insulin (pg/mL)	716 ± 316.7	819.7 ± 391.9	0.160	705.59 ± 468.8	599.6 ± 417.7	0.021
Glucagon (pg/mL)	79.1 ± 43.1	77.7 ± 40.0	0.921	65.2 ± 36.1	46.6 ± 30.8	0.004
A1c (%)	9.2 ± 1.2	6.9 ± 1.1	0.000	8.7 ± 0.9	6.9 ± 0.9	0.000
C-peptide (pg/mL)	1882.0 ± 690.1	2092.8 ± 956.2	0.193	1627.2 ± 585.6	1622.6 ± 609.6	0.059
GLP-1 pg/mL)	38.3 ± 25.3	45.5 ± 21.1	0.218	44.7 ± 38.7	83.8 ± 74,5	0.026
GIP (pg/mL)	39.1 ± 15.2	45.6 ± 39.0	0.201	38.9 ± 25.7	44.7 ± 34.8	0.405
TBAR (nmolMDA/mL)	8.9 ± 1.4	8.47 ± 0.9	0.262	9.0 ± 1.6	8.0 ± 0.7	0.035
TAOS	215.9 ± 34.5	264.3 ± 34.0	0.179	257.5 ± 61.3	205.3 ± 28.2	0.417

Data are means ± SD. GLP-1, Glucagon like peptide 1; *GIP* Gastric inhibitory polypeptide, *TBARS* Thiobarbituric Acid reactive substances, *TAOS* antioxidant state

Regarding the patients who used Vildagliptin, there was a significant decrease in insulin (*p* = 0.021), and glucagon levels (*p* = 0.004). There was significant increase in GLP-1 level (*p* = 0.026). There was a significant decrease in oxidative stress marker TBARS (*p* = 0.035). No difference was observed in TAOS (*p* = 0.417), GIP (*p* = 0.405) and C-peptide levels (*p* = 0.059) (Table 3).

## Discussion

For a long time, the association between cardiovascular disease and metabolic abnormalities during fasting state has been largely studied. However, more recently, metabolic abnormalities in postprandrial state have being recognized as important contributors to cardiovascular disease in T2DM and in other conditions associated with insulin resistance. Postprandial hyperglycemia has been reported as having a greater impact in cardiovascular disease and mortality than fasting hyperglycemia [14]. Others factors, like postprandial hyperlipidemia, have been also addressed as contributors to cardiovascular disease as assessed by several studies [15–21]. Clinical studies in T2DM with DPP-4 inhibitors provide evidences that incretin-based therapies improve fasting and postprandial glycemia and somewhat also dyslipidemia [22–25].

Our results confirm the benefits of vildagliptin, a DPP-4 inhibitor, in the HDL level of diabetic patients. Previous studies initially performed in rats [26] and subsequently in humans, demonstrated an increase in HDL in patients receiving vildagliptin [27]. This effect was observed at a dose of 50 mg/day, but it was greater when using 100 mg/day. This benefit occurs in both treatment-naïve patients, as in those that were already in use of metformin and had vildagliptin added [27–29]. In our study, at baseline there was no difference in HDL between the vildagliptin and gliclazida groups. After 16 weeks of follow up the patients in vildagliptin group showed better HDL in fasting and postprandial than the patients in gliclazide group. However, differently from literature data, we did not observed in our sample any differences in other lipid particles [27, 29].

The evaluation of patients’ weight between groups was hampered by a difference already in pre-treatment period, with a higher mean in the gliclazide group. Our data is agreement to the literature [30, 31]. We observed a positive variance (weight gain) in the gliclazide group and a small negative balance (weight loss) in vildagliptin group [32].

Assessing hormonal status of patients within each group before and after treatment, we were able to realize others benefits of vildagliptin. Besides the expected effects in increasing of GLP-1, we also observed significant changes in insulin and glucagon levels in Vildagliptin group.

The main mechanism of action of sulfonylureas through the ATP-sensitive potassium channels leads to increased insulin secretion, therefore were expected higher insulin levels following the introduction of gliclazide. The literature demonstrates that DPP4 inhibitors also lead to increased insulin due to improved beta-cell function, but we observed the opposite effect. Decreased insulin levels may be partially inferred from the reduction of insulin resistance with improved glycemic control [33–35].

The DPP4 inhibition by vildagliptin results in less degradation of GLP-1 leading to the expected effect in improving glycemic control. The decrease in glucagon levels, as observed only in vildagliptin group, is one of the important consequences caused by the increase of GLP-1. Such reduction diminishes the release of glucose mainly in the postprandial period. The intestinal hormone GIP also increased due to the inhibition of DPP4, however, in our sample this increase was insignificant. There was a decrease in TBARS in the group treated with vildagliptin, suggesting an additional benefit in the oxidative stress. This data is in accordance with literature regarding the effects of vildagliptin in oxidative stress [36, 37].

In conclusion, our study of 16 weeks with 36 T2DM showed some advantages in added vildagliptin instead of gliclazide to metformin in the treatment of uncontrolled hyperglycemia. Although both drugs effectively reduce A1c, vildagliptin has additional effects besides glycemic control. Patients treated with vildagliptin had a higher HDL at the end of the study, both fasting and postprandial. TBARS, a marker of oxidative stress, was reduced with vildagliptin treatment, suggesting some ability of this drug in modulating oxidative stress. Finally vildagliptin seems to reduce insulin resistance demonstrated by both reduction in serum levels of insulin and glucagon. To confirm those datas others studies with a longer period of treatment and a greater number of patient are necessary. It is also should be prudent to have studies with others oxidative stress parameters.
